# Some Physical Aspects of Cardiac Failure

**Published:** 1906-01-27

**Authors:** 


### SOME PHYSICAL ASPECTS OF CARDIAC FAILURE.

In a lecture upon this subject Dr. David Forsyth [1] observes that no treatment can be regarded as efficient which does not rest upon the triple foundation supplied by rest, the use of cardiac stimulants, and depletion, for the obvious indications are to diminish the calls upon the labouring heart, and to increase its capacity for dealing with the inevitable residue. The author urges the high importance of attention to means for alleviating the distresses attending the failing heart, for although the underlying affection is commonly a mortal one, the physician will have good cause to congratulate himself if he can rob the remaining days of the invalid's life of much of their pain and suffering.

Reduced to its simplest terms, the circulation consists of a force-pump, the heart, propelling liquid through a closed system of tubes. This force-pump is immensely assisted in the efficient performance of its duties by the suction of the negative pressure produced in the great veins by the act of inspiration. A third factor of immense importance in all problems connected with the circulation is the influence of gravity, and a fourth is the integrity of the splanchnic sympathetic nervous system, for the splanchnic veins are capable of accommodating all the blood in the body if their normal tension is deranged by failure of their sympathetic innervation.

A good example of the importance of physical considerations in the matter of heart affections is supplied by acute pericardial effusion, and it is with this disorder that the author is chiefly concerned. A case in point was that of a boy with a history of subacute rheumatic manifestations who came under observation for shortness of breath. He presented a slight extension of cardiac dulness, and the murmurs of double mitral disease. A week after his admission to hospital his cough became very troublesome, and resulted in the expectoration of much thin sputum coloured with bright blood. The note over the right apex in front became impaired, and auscultation over this area revealed many moist rales. From this date he went rapidly down-hill. The lungs were everywhere full of moist sounds, the area of the cardiac dulness still further encroached upon the lung, and the pulse became very rapid and feeble. The respirations rose to sixty, while the cough was constant and associated with attacks of hæmoptysis and cyanosis. Before death a pericardial friction sound became audible over the right ventricle. Post-mortem it was discovered that the pericardium was distended by six ounces of fluid, and the dilated heart showed evidence of old endocarditis. The right lung was solid, but not apparently from pneumonic consolidation; the appearance was that of an intense œdema. The left lung was similarly affected, but to a less degree.

This case points the moral of the physical considerations enunciated above. There is no doubt that the cause of death was the pericardial effusion, which, enclosed in the rigid pericardial sac, exercised a fatal mechanical influence upon the heart. The positive pressure thus appearing within the pericardial cavity led to compression of the thin-walled venæ cavæ, and obstructed the return of blood to the auricles. This obstruction was reflected in the pulmonary circuit by the œdema of the lungs, which again extended the vicious circle by inducing a persistent cough.

In the treatment of such a case two things are essential to success—namely, to relieve the mechanical embarrassment of the heart which is directly due to the bulk of the effusion, and to reduce the labours of the overburdened myocardium. In the early stages the former can be met by alterations in posture. In the first instance, a semi-recumbent posture will throw the weight of the pericardial contents upon the diaphragm, and thus increase the capacity of the chest at the expense of the abdomen. At a later stage, to turn the patient well over upon his left side will give considerable relief by throwing the intra-pericardial weight against the bony thorax. But when pressure symptoms become really obtrusive there is only one thing to be done—namely, paracentesis of the pericardium. Dr. Forsyth urges that this proceeding is in general too long postponed. The indications for the operation are, in his opinion: (1) Spreading cardiac dulness; (2) progressive enfeeblement of the pulse; (3) increasing dyspnœa, especially of the air-hunger variety; (4) distended veins; (5) cyanosis. But it is recommended that the presence of the first three of these indications is ample justification for paracentesis.

The most favourable spot for paracentesis of the pericardium is considered to be the left costo-xiphoid angle. The needle should be inserted here, and passed upward, backward, and slightly inward behind the sternum. The needle will then enter the pericardium at its lowest part after passing in for two or two and a half inches. In paracentesis of the pericardium it is necessary to regulate the speed with which the fluid is removed, for effusions in this situation often exist under a considerable degree of pressure, and rapid variations in the tension upon the heart are likely to affect the circulation profoundly.

[1] Clin. Jour., Jan. 17, 1906.

